# Integrated Native Mass Spectrometry Imaging of Soluble and Membrane Proteins

**DOI:** 10.1021/jacs.5c16821

**Published:** 2026-01-16

**Authors:** Oliver J. Hale, Helen J. Cooper

**Affiliations:** School of Biosciences, 1724 University of Birmingham, Edgbaston, Birmingham B15 2TT, U.K.

## Abstract

Native ambient mass spectrometry enables the analysis of intact protein complexes directly from fresh frozen tissue sections together with visualization of their spatial distribution as part of a mass spectrometry imaging workflow. Native mass spectrometry imaging typically employs nanospray-desorption electrospray ionization (nano-DESI), a liquid junction sampling approach. Imaging of both soluble and membrane proteins has been demonstrated by native nano-DESI but, crucially, imaging of one protein type has always been at the expense of the other, requiring tailored sample preparation and multiple tissue sections. Here, we introduce a new mode of nano-DESI operation that combines soluble and membrane protein analysis into a single experiment, requiring no sample preparation and only a single tissue section, and which is compatible with mass spectrometry imaging. Chromatography-like separation of soluble and membrane protein signals, observed as varying elution profiles, occurs when the nano-DESI probe is parked in a fixed location on the tissue. The elution profiles of proteins in both kidney and brain tissue were explored. The results show that elution profiles are quick to record, offer insight into the classification of unknown proteins detected from tissue and enable signal-to-noise improvements to imaging and native top-down mass spectrometry workflows.

## Graphical abstract

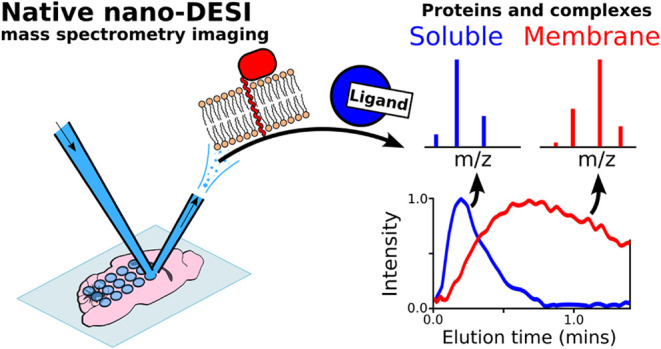

## Introduction

Native ambient mass spectrometry (NAMS) provides simultaneous spatial and structural information on proteins and their assemblies directly from tissue. To maintain the native protein structure as closely as possible, NAMS involves no, or very little, sample preparation: Ideally, fresh frozen tissue is simply sectioned onto glass slides and analyzed directly. For native ambient mass spectrometry imaging (MSI), tissue sections are typically sampled by nanospray desorption electrospray ionization (nano-DESI^1^), a liquid junction technique. We have previously demonstrated native MSI of protein assemblies,^2^ endogenous protein–ligand complexes,^3^ and protein-drug complexes formed in vivo.^4^ In each of these cases, the species imaged were soluble proteins.

Imaging of membrane proteins has been demonstrated by matrix-assisted laser desorption ionization (MALDI) MSI,^5^ but it requires substantial sample preparation and does not produce ions amenable for on-tissue top-down MS (i.e., they are predominantly singly charged).^6^ Furthermore, while pH-stable, strongly binding protein complexes have been detected by MALDI MS,^7–9^ the sample conditions do not mimic physiological conditions and therefore do not preserve protein complexes in their native,^10^ folded state for analysis in the gas phase.

We have separately developed imaging of membrane proteins under nondenaturing conditions using nano-DESI MSI.^11,12^ Initially, we showed native MSI of a membrane protein assembly, aquaporin-0, directly from eye lens tissue by incorporating a mass spectrometry-compatible detergent into the nano-DESI solvent system at a level above the critical micelle concentration (CMC).^11,13^ The abundance and substantial *m*/*z* difference of aquaporin-0 compared to other abundant proteins in the eye lens made this membrane protein readily detectable. However, when this approach was applied to other tissues, membrane protein signals were masked by the signals of abundant, soluble proteins. To address this issue, we developed a washing procedure using aqueous ammonium acetate prior to nano-DESI MS analysis. This procedure depleted soluble proteins from the tissue while leaving the spatial distribution of lower solubility membrane and membrane-associated proteins undisturbed.^12^

The washing approach enriches for membrane proteins at the expense of soluble proteins, meaning two experiments, and two separate tissue sections, are necessary to co-image these two protein types, with consequences such as the introduction of artifacts and spatial anomalies during image registration. Furthermore, the introduction of a sample preparation step introduces risks for experimental error, e.g., disruption of tissue structure, critical to avoid when working with rare samples. A far preferable approach would be simultaneous imaging of both protein classes in a single imaging experiment. One possible answer is online separation, either gas- or solution-phase, of proteins post-sampling.

The advent of ion mobility spectrometry technologies coupled to MS has resulted in the widespread implementation of millisecond-scale gas-phase separations in MSI workflows for metabolites,^14,15^ lipids,^16–18^ carbohydrates^19^ and intact soluble proteins.^20–22^ Solution-phase separation techniques, such as chromatography and electrophoresis, are not usually associated with MSI due to their time scales (minutes to hours for effective separation) and their incompatibility with the most prevalent MSI techniques (MALDI,^23,24^ DESI,^25^ and SIMS^26^). An exception that integrates solution-phase separation is the development of a nano-DESI-inspired liquid junction and gas-pumped electrospray system coupled to capillary electrophoresis. That system enabled electrophoretic separation of metabolites sampled directly from tissue and blood^27,28^ and was subsequently developed to add MSI functionality.^29^ That work highlights the unique advantages of nano-DESI over MALDI, DESI, and SIMS imaging modalities: the use of a solvent sampling system, separated from the ionization event, can be adapted into powerful analytical methods.

Nano-DESI MSI is usually performed in a continuous mode where the tissue surface is scanned in lines (herein referred to as “continuous mode”) underneath the sampling probe. The linescans are subdivided into pixels during postprocessing, allowing the generation of ion images. Here, we interrogate an alternative “discrete” mode, which parks the nano-DESI probe in a fixed location on the tissue surface for a defined period before moving to the next location. Importantly, although the pixel sizes in the final ion images are the same for both modes, discrete-mode nano-DESI allows molecules from the tissue to be dissolved over time, whereas in continuous mode the time in which the liquid junction is in contact with the tissue is dictated by the speed at which the probe is rastered. Our results show that this feature enables inherent differences in protein solubility to be exploited to separate soluble, membrane-associated and membrane protein molecules in a chromatography-like manner prior to ionization. Discrete-mode native nano-DESI enables soluble, membrane-associated and transmembrane proteins to be analyzed directly from the same tissue section in a single experiment without sample preparation.

## Experimental Section

### Materials

MS-grade water was purchased from Fisher Scientific. HPLC-grade ammonium acetate was bought from J.T. Baker (Deventer, The Netherlands). C_8_E_4_ detergent was obtained from Merck (Gillingham, UK). Helium gas (99.996% purity) and nitrogen gas (>99.995%) were obtained from BOC (Guildford, UK). FlexMix calibration solution and fused silica tubing were purchased from Thermo Fisher Scientific (Waltham, MA).

### Tissues

Fresh frozen rat kidneys were a gift from Prof. Richard Goodwin (AstraZeneca). Fresh frozen brains from wild-type mice were a gift from Dr. Richard Mead (University of Sheffield, UK). Whole, fresh sheep eyes were purchased from DissectUK (Birmingham, UK). Eyes were dissected, the lenses extracted, placed on aluminum foil, and snap-frozen in liquid nitrogen. Whole kidneys were sectioned into 10 μm-thick sections in the sagittal plane with a CM1810 cryotome (Leica Microsystems, Wetzlar, Germany). Similarly, 10 μm-thick sagittal cryosections of brain were prepared from brains bisected down the midline. Eye lens tissue was sectioned at 20 μm. Tissue sections were thaw-mounted to glass microscope slides and stored at −80 °C until analysis. For most analyses, the tissues were defrosted in a desiccator prior to analysis but were not prepared further. The protocol published previously^12^ was applied for comparison experiments in which discrete-mode nano-DESI was evaluated against tissue washing for membrane protein enrichment. In short, whole tissue sections (kidney and brain) were washed by submerging in 200 mM aqueous ammonium acetate for 1 min, followed by 10 min of drying under vacuum. The wash-and-dry cycle was performed three times before analysis by nano-DESI.

### Nano-DESI Ion Source

The nano-DESI ion source is home-built and has been described previously.^3^ Briefly, the source consists of an XYZ stage that moves the sample underneath a nano-DESI probe that is fixed in position. The probe consists of two fused silica capillaries at an angle 60° incident to the sample surface: the first delivers solvent from a syringe to the sample, and the second aspirates the solvent and dissolved analytes into the mass spectrometer ion inlet. A high voltage is applied to the solvent to initiate electrospray ionization. The nano-DESI solvent system was 200 mM aqueous ammonium acetate with C_8_E_4_ detergent added to approximately 2× the critical micelle concentration (0.5% by volume) to enable membrane protein analysis. This solvent was delivered by syringe pump at a rate of 0.65 μL/min. The nano-DESI liquid junction was tuned to approximately 100 μm in diameter for all experiment types.

### Mass Spectrometry

For MS analysis of kidney and brain tissue, the nano-DESI ion source was attached to an Orbitrap Eclipse mass spectrometer (Thermo Fisher Scientific) equipped with the HMR^n^, ETD, and proton transfer charge reduction (PTCR) options. Ion source conditions were: electrospray voltage = 1125 V, ion transfer tube temperature = 275 °C, source pressure ∼2.3 Torr, source collision voltage = 120 V, and the source CID scaling factor = 0.018. The mass spectrometer was run in “high pressure mode” (ion routing multipole (IRM) = 20 mTorr) with a selected ion monitoring method (SIM, using linear ion trap isolation and orbitrap detection) over *m*/*z* 2400–4000, an automatic gain control target of 5 × 10^6^ charges, an injection time of 750 ms, orbitrap analyzer resolution set to 7500 (fwhm at *m*/*z* 200, transient length = 16 ms), and three transients (“microscans”) averaged per scan. Note that this relatively low orbitrap resolution setting is advantageous for native MS analysis of intact proteins. Low-resolution settings show improved intact protein signal-to-noise ratio compared with high-resolution settings, which require protein ions to survive image current recording over a long duration (e.g., ≥ 512 ms transient for resolution of 240,000 at *m*/*z* 200).^30,31^

For MS^2^ analysis of eye lens proteins, the nano-DESI ion source was attached to an Orbitrap Ascend Structural Biology mass spectrometer (Thermo Fisher Scientific) equipped with the native MS, ETD, PTCR, and ultraviolet photodissociation (UVPD) options. Ion source conditions were as follows: electrospray voltage = 1250 V, ion transfer tube temperature = 275 °C, source pressure ∼2.45 Torr, source collision voltage = 120 V, and the source CID scaling factor = 0.045. The mass spectrometer was operated in “intact protein” and “high pressure mode” (front IRM = 15 mTorr, back IRM = 20 mTorr). The quadrupole mass filter was used for ion isolation up to *m*/*z* 8000, and collisional activation (HCD MS^2^) was performed in the front IRM. For PTCR MS^2^, the reagent anion target was set to 5.0 × 10^5^ charges. The orbitrap resolution was set to 7500 (fwhm at *m*/*z* 200). Full profile mode was turned on for all eye lens analysis.

Proteins detected in this work were either identified here by HCD MS^n^ or were previously identified by MS^n^ and assigned here based on their intact molecular weight. Proteins in rat and mouse tissue were identified using a nano-DESI ion source attached to the Orbitrap Eclipse described above. The linear ion trap was used for ion isolation, and the IRM was set to 20 mTorr with N_2_ collision gas. An orbitrap resolution of 240,000 (fwhm at *m*/*z* 200) was used for product ion detection. A summary of proteins in this work is provided in Table S1, Supporting Information. Specific MS^n^ details, such as normalized collision energy (NCE) and isolation width, are included in relevant figure captions for each protein. MS^n^ product ion spectra were searched against reviewed proteoforms in the mouse (UP000000589) or rat (UP000002494) proteomes using Prosight PC (v4.1). Intact proteoforms were searched with a tolerance of 1 kDa to allow for PTMs and noncovalent ligands, and product ions were assigned within a tolerance of 20 ppm. Confident protein identification was guided by the preferential formation of specific product ions (i.e., C-terminal to Asp and N-terminal to Pro) during native TDMS analysis.^32^

### Mass Spectrometry Imaging

In the initial evaluation of discrete-mode nano-DESI, tissue locations were sampled for 3 min. For subsequent discrete-mode nano-DESI MSI, consecutive tissue locations were sampled for 1.5 min, controlled by a custom LabVIEW program. Pixel size was set to 100 × 200 μm. MS image files were generated by processing Thermo.raw files with Firefly (v. 3.2.0.23, Prosolia Inc.) using either the time binning function (for continuous mode) or the spot sampling function (for discrete mode).

For continuous-mode nano-DESI MSI, images were acquired as linescans at a rate of 5 μm/s, resulting in the transit of the liquid junction over a specific location in 20 s. This movement rate allows for lower-solubility proteins to be detected in all experiments. Pixel size was set to 94 × 200 μm.

### Multivariate Image Analysis

Thermo.raw files were converted to mzML by MSConvert (v3.0, ProteoWizard).^33^ A 4D imzML file (x, y, elution time, and *m*/*z* dimensions) was then constructed from the mzML files using a custom Python script (available from https://github.com/coopergroup-massspec). Within the same Python script, spectra in the imzML file were then subjected to binning (width = 2 *m*/*z*), normalization, principal component analysis (PC = 5), and k-means clustering (clusters = 2) to uncover image pixels within the two major brain tissue classes: gray matter and white matter. Mean mass spectra for the two classes were generated from 369 and 51 pixels, respectively. An image visualizer within the script enabled filtering of *m*/*z* images by elution time. Example spectra and *m*/*z* images were generated with an elution time width = 3 scans, approximately = 7.8 s.

## Results and Discussion

### Elution Profiles Separate Proteins by Solubility

Empirical observations in our laboratory suggested that proteins sampled during discrete-mode nano-DESI analysis exhibited chromatography-like elution profiles, with some protein signals detected earlier in the sampling time scale and some later. To understand the root of these observations, five discrete, adjacent locations were sampled on rat kidney and mouse brain (Figure 1). Each location was sampled for 3 min before the probe was moved to the next location. This sampling duration per location was sufficient to understand signal decay over time and guided the decision to reduce acquisition time to a more practical 1.5 min/pixel for imaging experiments (below). The total ion chromatograms (TICs) indicated the majority of protein signal was detected over the first minute of sampling, but some signal was detected throughout. Examination of extracted ion chromatograms (XICs) for individual proteins and complexes revealed distinct elution profiles for each species. Notably, the most soluble proteins (e.g., SOD1, Arf3) exhibited an intense signal for the first 30 s, which was rapidly depleted thereafter. Conversely, proteins associated with the cell membrane via lipid anchors (e.g., GDP-bound Rab3a) or integral membrane proteins (e.g., cytochrome B5, VDAC1) exhibited broader elution profiles with their peak intensity occurring later in the profile.

**Figure 1:**
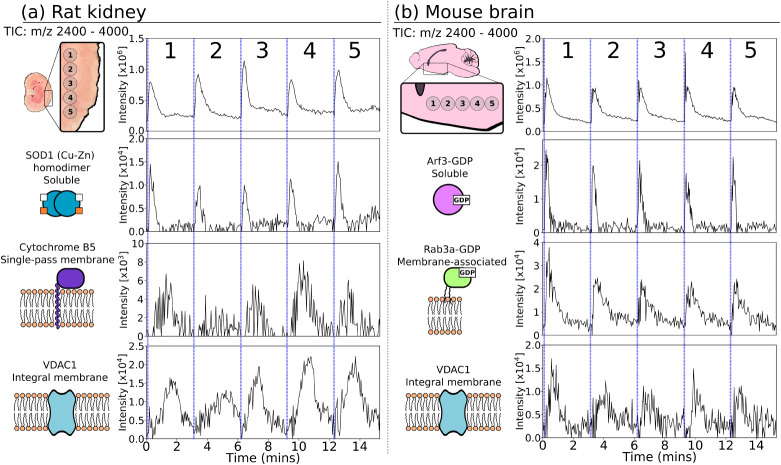
(a) Total ion chromatogram (TIC) and extracted ion chromatograms (XIC) for proteins in rat kidney; TIC (*m*/*z* 2400–4000), SOD1 homodimer in complex with 4-metal ions (*m*/*z* 2900.3^11+^), Cytochrome B5 (*m*/*z* 2545.3^6+^, *m*/*z* 3054.1^5+^), VDAC1 (*m*/*z* 2788.9^11+^, *m*/*z* 3067.9^10+^, *m*/*z* 3408.5^9+^, *m*/*z* 3834.6^8+^). (b) TIC and XICs for proteins in mouse brain: TIC (*m*/*z* 2400–4000), Arf3-GDP complex (*m*/*z* 2641.3^8+^, 3018.6^7+^, 3421.1^6+^), Rab3a-GDP complex (*m*/*z* 2589.3^10+^, 2876.8^9+^,3236.4^8+^), VDAC1 (*m*/*z* 3067.6^10+^, 3408.5^9+^,3834.2^8+^). Each tissue was sampled at 5 adjacent locations for 3 min per sample. XIC tolerance = ± 0.15 *m*/*z*. Sampling spot diameter ∼100 μm.

### Discrete-Mode Nano-DESI for Integrated Imaging of Soluble and Membrane Proteins

Based on the findings above, we compared discrete-mode and continuous-mode nano-DESI protein imaging. The difference in sampling mode is evident on the tissue surface postanalysis (Figure 2). Three imaging workflows were compared: (i) discrete-mode nano-DESI of unwashed tissue; (ii) continuous-mode nano-DESI of unwashed tissue; (iii) continuous-mode nano-DESI of tissue previously washed with 200 mM aqueous ammonium acetate to deplete soluble protein signal. In all cases, the nano-DESI solvent system was tailored for membrane protein analysis, i.e., 200 mM ammonium acetate +2× CMC of C8E4 detergent. Each imaging workflow was applied to separate, adjacent sections of mouse brain. For reference, the discrete-mode image required ∼12 h to complete. In comparison, each continuous-mode image covering the same tissue surface area was ∼2.4 h in duration. These approximations include all “dead time” that occurs during analysis due to lifting and repositioning of the probe, stabilization of the liquid junction, and reinstating of tissue contact at each pixel location (for discrete mode, ∼1.4 h/image) or between line scans (for continuous mode, ∼0.05 h/image).

**Figure 2:**
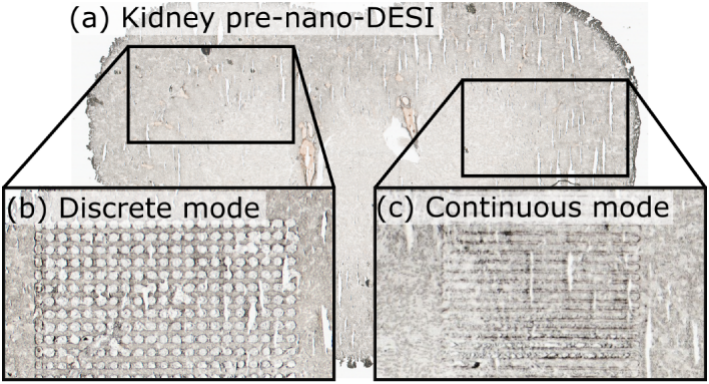
(a) Optical image of a rat kidney section prior to nano-DESI sampling. Highlighted regions of the kidney display (b) the sampling points of a discrete-mode nano-DESI MSI experiment and (c) the linescans of a continuous-mode nano-DESI MSI experiment.

The ion images generated from multiple charge states of each protein or complex from these experiments are shown in Figure 3, together with elution profiles for the discrete-mode analyses. The images for soluble protein complexes ADP-ribosylation factor 1 (Arf1+GDP; charge states 8^+^, 7^+^), ADP-ribosylation factor 3 (Arf3+GDP; 8^+^, 7^+^), and carbonic anhydrase II (CAH2+Zn^2+^; 11^+^ – 9^+^) are comparable between discrete-mode and continuous-mode (unwashed), with the Arf complexes showing abundance in the hippocampus and CAH2 abundant in the corpus callosum. Continuous-mode images of the washed tissue show the spatial distribution of these soluble proteins has been disrupted and their signal depleted. An outlier to this observation was the soluble cytoplasmic protein complex SIRT2.2+Zn^2+^ ((14^+^ – 12^+^) identified by native top-down MS in Figure S1,Table S2, Supporting Information), which exhibited a well-defined, unperturbed spatial distribution after tissue washing that correlated with that observed in unwashed continuous-mode. SIRT2.2+Zn^2+^ was found to have an elution profile more like the membrane-associated and transmembrane proteins. This short isoform of SIRT2 is known to have a strong association with myelin.^34,35^ Its extraction requires the myelin membrane to be dissolved for its release, hence the extended elution profile.

**Figure 3:**
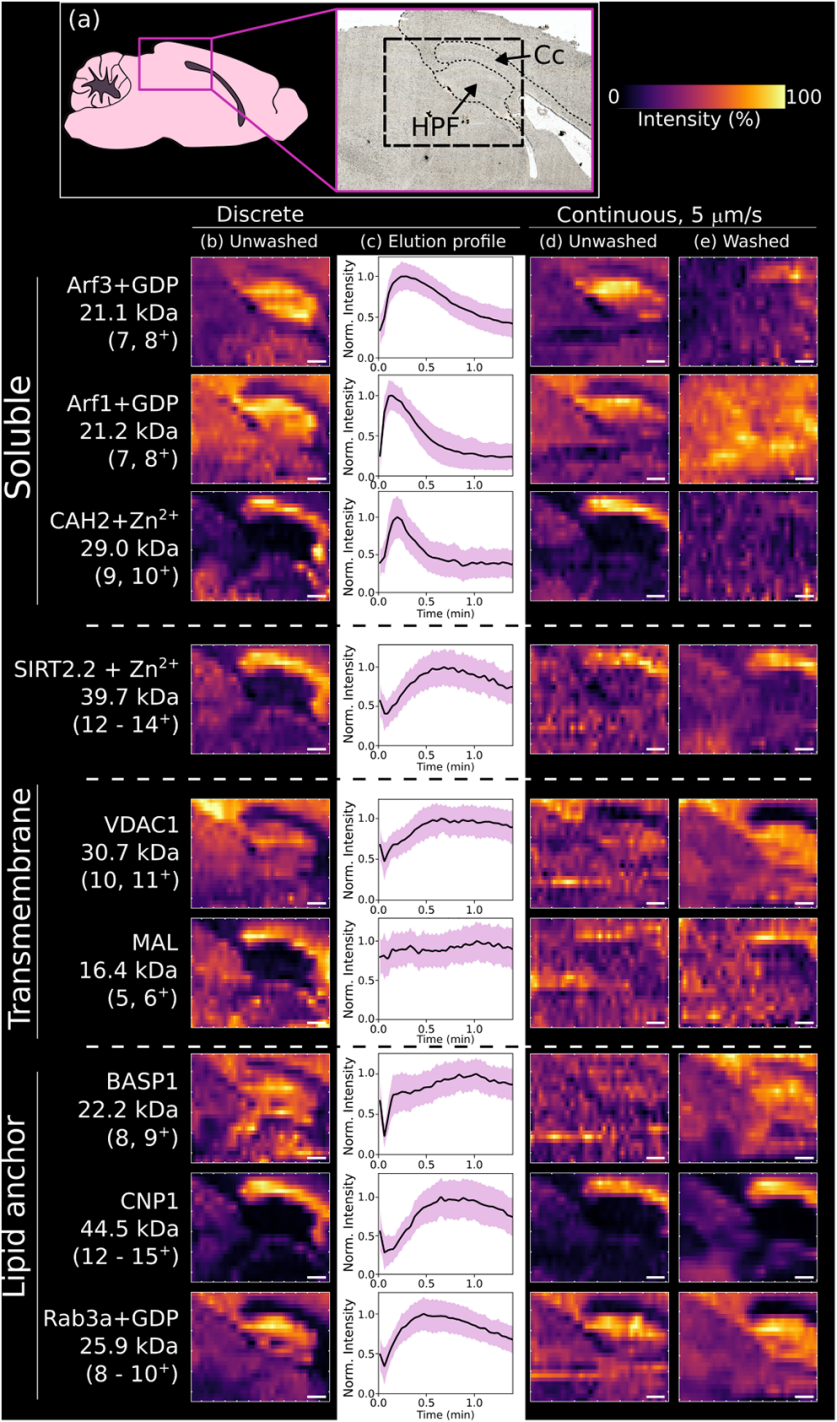
Evaluation of discrete mode versus continuous modes of nano-DESI with unwashed and washed tissue. (a) Depiction of the sampled region in the mouse brain (*Cc* = corpus callosum, HPF = hippocampal formation). (b) Nano-DESI MS images for proteins acquired using the discrete mode. (c) Elution profiles for proteins in (b). Each elution profile is the average of 390 pixels from the images in (b) for the specified charge states with tolerance = 60 ppm, summed and intensity normalized. The purple band indicates ± 1 standard deviation. (d) Nano-DESI MS images for proteins acquired using the continuous mode without tissue washing and (e) with tissue washing. Multiple charge states for each protein comprise each image (specified in Figure). Image scale bar = 400 μm.

The spatial distributions of transmembrane proteins Mt-VDAC1 (12^+^–9^+^, Figure S2, Table S3, Supporting Information) and myelin and lymphocyte protein (MAL, 6^+^, 5^+^, Figure S3, Table S4, Supporting Information) were best resolved with discrete-mode and continuous-mode (washed). Definition of the corpus callosum was lost in the continuous-mode (unwashed). The image for MAL is notably best in discrete mode and features a relatively flat elution profile, suggesting this protein benefits from a greater number of scans for which to build in signal. This elution profile also suggests why MAL images in continuous mode are poor regardless of tissue washing—the protein might simply require time to be dissolved, and continuous mode does not allow for that. It is possible that a longer sampling period would further benefit proteins such as MAL, though generally it was observed that after 3 min most protein signal was depleted.

Membrane-associated proteins and complexes featuring lipid anchors exhibited intermediate behavior. The BASP1 (9^+^, 8^+^, Figure S4, Table S5, Supporting Information) image from continuous mode (unwashed) was poorly defined, and continuous mode with washing resulted in a blurred image. The discrete-mode image was the best resolved. Other membrane-associated proteins, including CNP1 (15^+^–12^+^, a proteoform featuring N-terminal acetylation, C-terminal truncation and C-terminal S-geranylgeranylation, Figure S5, Table S6, Supporting Information) and Rab3a+GDP (10^+^–8^+^, a proteoform featuring two S-geranylgeranylations as well as a noncovalently bound GDP molecule, Figure S6, Table S7, Supporting Information), showed good image resolution across all experiment types. The membrane-associated protein elution profiles are extended as observed for the integral membrane proteins indicating their lower relative solubility. CNP1 is associated with the myelin sheath by lipid anchor, hence the spatial localization to the corpus callosum. Rab3a was most abundant in the hippocampus. BASP1 relies on myristoylation for interaction with the membrane, so it likely has the weakest interaction of these three proteins, which results in its disrupted spatial distribution from washing. The UniProt entry for CNP1 reports the C-terminal lipid PTM as S-farnesylation, but this was inconsistent with the MW difference measured here. Rather, the MW difference (286 Da) is consistent with S-geranylgeranylation and C-terminal methylation of the C-terminal cysteine residue. Further, the unprocessed sequence of CNP1 features a CaaX motif (CTII) ideal for geranylgeranylation.^36^ Similarly, the Rab3a-GDP complex contains two C-terminal geranylgeranylated cysteine residues, resulting in a MW increase of ∼558 Da (2× geranylgeranylation + C-terminal methylation) compared to the unmodified amino acid sequence. Geranylgeranylation results in a stronger interaction with the membrane than myristoylation, and multiple PTMs further increase the interaction strength. For this reason, CNP1 and Rab3a are less disrupted by tissue washing.

### Elution Time Filtering for MS Imaging

Mass spectra at different time points in the elution profile exhibit noticeably different signals (Figure S7, Supporting Information). Relatedly, ion images can be generated for defined elution time ranges, enabling improvements to image specificity and S/N in a similar manner to ion mobility filtering used previously.^16,22^ To demonstrate, images for four proteins abundant in the corpus callosum are shown in Figure 4. Images were generated for two distinct elution time ranges, separating more and less soluble proteins. Being soluble, CAH2+Zn^2+^ (Figure 4a) has a well-defined image for 0.0 – 0.3 min elution time but is depleted for 0.7 – 1.5 min elution time, resulting in an image with poor S/N. The inverse is true for SIRT2.2+Zn^2+^ (Figure 4b), VDAC (Figure 4c) and CNP1 (Figure 4d) since these proteins exhibit low solubility. Technological barriers notwithstanding, an increase in the elution profile resolution would result in images with increased specificity, potentially allowing the separation of proteoforms by their lipid posttranslational modification, e.g., for BASP1 and CNP1 discussed above.

**Figure 4:**
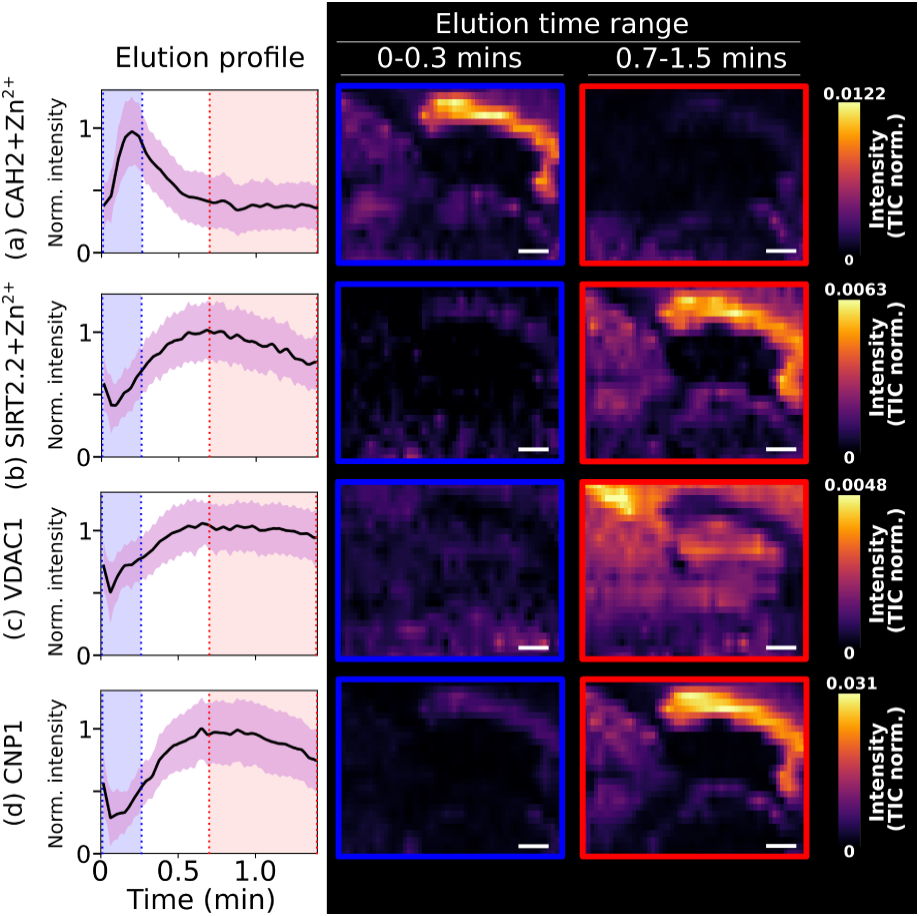
Discrete-mode nano-DESI images for proteins abundant in the corpus callosum produced from two distinct elution time ranges: 0.0–0.3 min (blue) and 0.7–1.5 min (red). (a) CAH2+Zn^2+^ (soluble), SIRT2.2+Zn^2+^ (soluble, myelin-associated), (c) VDAC (multipass transmembrane protein), and (d) CNP1 (membrane-associated via lipid anchor). Images for each protein are shown on the same normalized intensity scale. Scale bar = 400 μm.

To further interrogate the time-resolved imaging data set, principal component analysis followed by K-means clustering was performed (Figure S8, Supporting Information). This analysis revealed the major source of variance was tissue type (white or gray matter) rather than elution time. Mean spectra from the two tissue types are shown in Figure 5a (gray matter) and b (white matter), with corresponding elution profiles shown in Figure 5c and d. These elution profiles have improved signal-to-noise when compared with the full data set profiles in Figure 3 due to a lower contribution from background signal. The effect is especially evident with MAL, which exhibited a relatively flat elution profile in Figure 3 but has a much clearer extended elution profile in Figure 5d. This analysis also reveals that distinct elution profiles exist even between ostensibly soluble proteins. In gray matter, GDP-bound Arf3 eluted rapidly, while GDP-bound Arf1 eluted over a broader time range despite both interacting with membranes in a transient manner via N-terminal myristoylation. Their differing elution profiles likely result from the stronger interaction with membranes of Arf1 than Arf3.^37^ Similarly, CAH2 exhibited a broader elution profile than PEBP1 in the white matter. As with SIRT2.2, this could be indicative of the partitioning of these proteins to specific tissue subtypes within tissue macrostructures.

**Figure 5:**
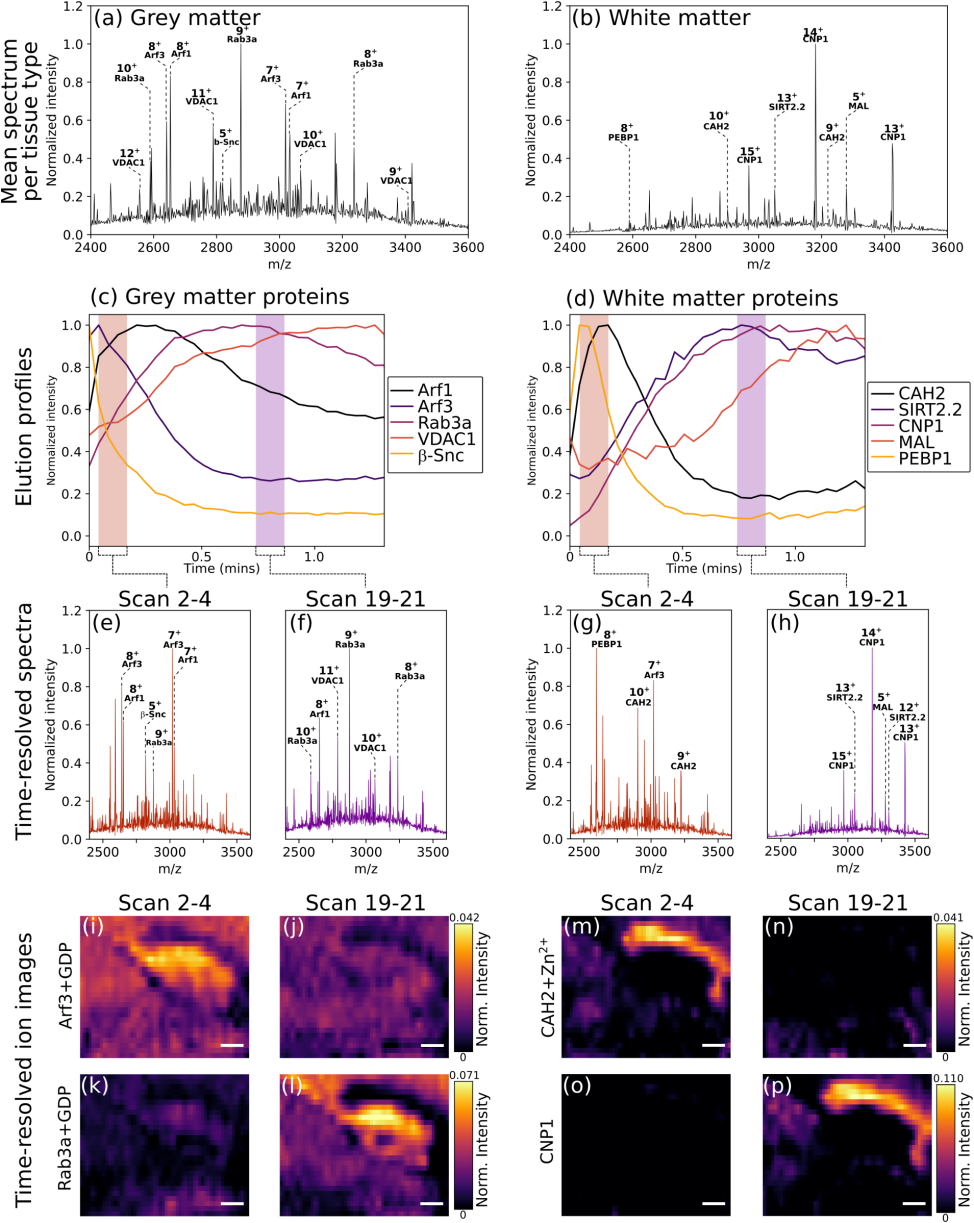
Mean mass spectra for gray matter (369 pixels) (a) and white matter (51 pixels) (b) grouped by PCA and k-means clustering. Elution profiles for proteins abundant in the (c) gray matter (Arf1+GDP; 7^+^, 8^+^, Arf3+GDP; 7^+^, 8^+^, Rab3a+GDP; 8^+^–10^+^, VDAC1; 10^+^, 11^+^, & β-synuclein; 5^+^) and (d) white matter (CAH2+Zn^2+^; 9^+^, 10^+^, SIRT2.2; 12^+^–14^+^, CNP1; 12^+^–15^+^, MAL; 5^+^, 6^+^ & PEBP1; 8^+^). Time-resolved mass spectra for gray matter pixels averaged for time scans (e) 2–4 (approximately 5.2–13.0 s) and (f) 19–21 (approximately 49.4–57.2 s). Time- resolved mass spectra for white matter pixels (g) time scans 2–4 and (h) 19–21. Time-resolved ion images for the scan ranges noted above for (i, j) Arf3+GDP complex, (k, l) Rab3a+GDP complex, (m, n) CAH2+Zn^2+^ complex, and (o, p) CNP1. Ion image pairs for each protein are displayed with the same intensity scale. Charge states in images are as for the elution profiles. Image scale bar = 400 μm.

The above analysis enables the generation of focused time-resolved images (compared with the coarse-grain time-resolved images shown in Figure 4). Figure 5i-l show images for GDP-bound Arf3 and GDP-bound Rab3a obtained at their maximum elution (corresponding to scans 2–4 (5.2–13.0 s) and scans 19–21 (49.4–57.2 s)) with associated time-resolved mean mass spectra (gray matter) in Figure 5e and f. Similarly, Figure 5m-p show time-resolved images of Zn^2+^-bound CAH2 and CNP1, both of which are localized to the corpus callosum. Time-resolved mean mass spectra (white matter) are shown in Figure 5g and h.

### Elution Time Filtering Reduces Spectral Complexity for MS/MS

Discrete-mode nano-DESI also has benefits for tandem mass spectrometry analysis of protein complexes from crude samples. As an example, eye lens tissue was sampled by nano-DESI. Eye lens is rich in soluble and membrane protein assemblies. After inspection of the full-scan mass spectrum (Figure S9, Supporting Information), *m*/*z* 6281 ± 15 was isolated using the quadrupole mass filter for MS^2^ analysis. PTCR MS^2^ revealed at least three overlapping proteins and/or complexes coisolated within this isolation window, including the 16+ charge state of the soluble β-(B2)_2_A1A4-crystallin heterotetramer and the 18+ charge state of the homotetrameric membrane protein Aquaporin-0 (Aqp0) (Figure S10, Supporting Information). These assemblies were previously identified by nano-DESI native top-down MS.^11,38^ Clearly, a product ion spectrum resulting from collisional activation of ions in this *m*/*z* 6281 ± 15 window would be chimeric, i.e., containing product ions from multiple precursors. Five discrete nano-DESI positions were sampled on the eye lens tissue with a residence time of ∼1.3 min. The mass spectrometer was set to collisionally activate *m*/*z* 6281 ± 15 (HCD, 70 V) over the time course. For the initial period of contact with the lens tissue (∼0.2 – 0.5 min), the HCD MS^2^ spectrum (Figure 6a) contained signals for multiple crystallin monomers and Aqp0 monomers and trimers, the result of dissociation of higher-order complexes. Conversely, the HCD MS^2^ spectrum from the period 0.59–1.5 min (Figure 5b) contained only signals for the Aqp0 dissociation products. Elution profiles for the product ions revealed that product ions derived from soluble (i.e., crystallins) and membrane (i.e., Aqp0) protein complexes were detected over different periods owing to their precursor solubility (Figure 5c-g). Discrete-mode nano-DESI therefore enables otherwise chimeric tandem mass spectra featuring soluble and membrane proteins to be simplified by elution time filtering. Furthermore, the monitoring of MS/MS product ions offers increased specificity over the intact protein *m*/*z*, which have higher likelihood of overlap with other chemical signals.^39^ Future elution profile methods may default to monitoring product ions for the increased confidence they offer.

**Figure 6:**
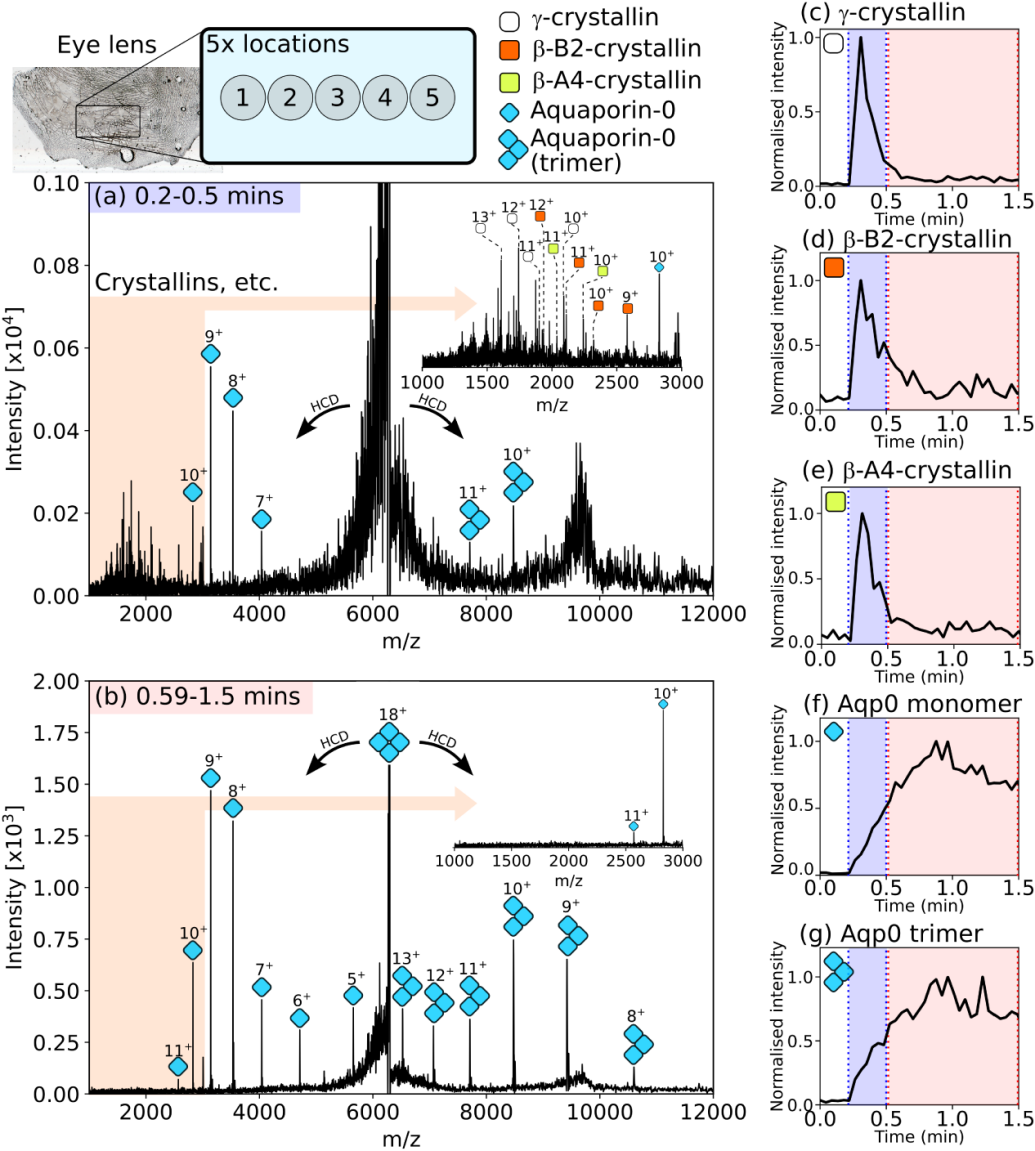
Discrete-mode nano-DESI-HCD MS^2^ of *m*/*z* 6281 ± 15 (HCD voltage = 70 V). Contact with the tissue was made ∼0.2 min after data acquisition was initiated. (a) Representative mass spectrum from location 1 for the period 0.2 – 0.5 min showing a chimeric product ion mass spectrum of crystallin and Aqp0 subunit ions ejected from higher-order complexes. (b) Product ion mass spectrum (location 1) for the period 0.59 – 1.5 min. Only Aqp0 signals were detected. MS^2^ product ion elution profiles for crystallin and Aqp0 subunits: (c) γ-crystallin (10^+^–13^+^), (d) β-B2-crystallin (9^+^–12^+^), (e) β-A4-crystallin (10^+^–12^+^), (f) Aqp0 monomers (5^+^–11^+^) and (g) Aqp0 trimers (8^+^–13^+^). Elution profiles are the average of 5 discrete sampling locations and generated using the charge states noted.

## Conclusions

A new, discrete sampling mode for nano-DESI MS, which enables co-imaging of soluble and membrane-intact proteins and protein complexes and aids in the simplification of chimeric MS^2^ spectra, has been developed. Proteins and complexes can now be characterized by their elution profile, which indicates their solubility and offers advantages for improving the specificity of MSI and MS/MS methods. Discrete-mode nano-DESI MSI alleviates issues encountered with continuous-mode MSI, namely poor detection of membrane proteins without tissue prewashing and the disruption of soluble protein distributions with prewashing. The case of SIRT2.2+Zn^2+^ exhibiting an extended elution profile owing to the tissue environment in which it resides is important insight for developing methods to improve the proteomic depth of tissue analysis by nano-DESI MS. The elution profiles reported here are presumed to be specific to the solvent system used. That is, it should be expected that other solvent systems and mass spectrometry-compatible detergents will result in the solubilization of different proteins at different rates—and perhaps different proteins altogether. This aspect will be investigated in a follow-up study.

The greatest drawback of the discrete-mode nano-DESI MSI for protein imaging is the extended period required for sampling each pixel. To take advantage of the elution profile, a per-pixel time of ∼1 min is necessary compared to ∼20 s for an equivalent pixel in continuous-mode experiments conducted here. As discussed for images here, this resulted in an image acquisition time of ∼12 h for discrete mode versus ∼2.5 h for continuous mode. This effect is not limited to this technique; for example, MSI coupled with trapped ion mobility separation also suffers from extended experiment times in exchange for richer molecular information.^16^ The ability to run a single experiment combining analysis of soluble and low-solubility proteins somewhat mitigates the extended experiment time, and we expect that optimizations to run time will be possible through improvements to ion source control software, for example. Future acquisition of MS images with high spatial resolutions will be particularly impacted, as doubling the spatial resolution will result in a 4-fold increase in pixels to acquire and a corresponding 4-fold increase in acquisition time. The spatial resolution of discrete-mode nano-DESI MSI is also tied explicitly to the liquid junction size, which is not the case in continuous mode, where the leading edge effect of the moving liquid junction results in higher resolving powers.^40,41^ Thus, advancing to higher spatial resolutions for discrete-mode nano-DESI MSI will require methods for precise control of liquid junction size.

## Supplementary Material



## Notes

Mass spectra, HCD MS^2^ and MS^n^ spectra, PTCR MS2 spectra, details of sequence ions observed in MS^2^ and MS^n^ experiments, summary of proteins detected, details of 4D imzML file processing, and multivariate analysis (PDF)
Data processing scripts are available via GitHub: https://github.com/coopergroup-massspec. Supplementary data supporting this research is openly available from https://doi.org/10.25500/00001511.
