# Supplementary material for: Integrated Native Mass Spectrometry Imaging of Soluble and Membrane Proteins
Source: J Am Chem Soc. 2026 Jan 16;148(3):3185–93. doi: 10.1021/jacs.5c16821 (PMC12856882; doi:10.1021/jacs.5c16821)
Supplement: Supplementary file 1 [file ja5c16821_si_001.pdf]

Supporting information for “Integrated native mass spectrometry imaging of soluble and membrane proteins”

Oliver J. Hale and Helen J. Cooper*

School of Biosciences, University of Birmingham, Edgbaston, Birmingham B15 2TT, UK.

*To whom correspondence should be addressed: h.j.cooper@bham.ac.uk

Table of Contents: Figures

Figure S1: nano-DESI-HCD MS2 mass spectrum for m/z 3051^{13+}. S3
Figure S2: nano-DESI-HCD MS2 spectrum for mouse Mt-VDAC1, m/z 3067^{10+}. S5
Figure S3: nano-DESI-HCD MS2 spectrum of m/z 3279^{5+}.......... S6
Figure S4: nano-DESI-HCD MS2 spectrum of m/z 2771^{8+}.......... S7
Figure S5: nano-DESI-HCD MS2 mass spectrum for m/z 3181.4^{14+}. S9
Figure S6: nano-DESI-HCD MSn of Rab3a.. .... S11
Figure S7: representative mass spectra for three elution time ranges from two different pixels representing grey (pixel 37) and white (pixel 76) brain matter S12
Figure S8: 4D imzML file processing and multivariate analysis S13
Figure S9: nano-DESI full scan mass spectrum of protein complexes in the eye lens S14
Figure S10: nano-DESI-PTCR MS2 of m/z 6281±15.......... S15

Table of Contents: Tables

Table S1: Summary of endogenous proteins detected in this work. S2
Table S2: SIRT2.2 sequence ions. S4
Table S3: Mt-VDAC1 sequence ions. S5
Table S4: sequence ions for MAL. S6
Table S5: sequence ions for BASP1. .... S8
Table S6: sequence ion table for 2',3'-cyclic-nucleotide 3'-phosphodiesterase (CNP1). S10
Table S7: sequence ions for Rab3a. S11

Table S1: Summary of endogenous proteins detected in this work.

Protein	Organism	Uniprot entry	Protein type	ID(DOI)a
**SOD1 (dimer)**	Rat	P07632	Soluble protein-metal complex	10.1039/D3SC04933G
**Cytochrome B5**	Rat	P04166	Single-pass membrane	10.1021/jacs.3c03454
**VDAC1**	Rat	Q9Z2L0	Beta barrel integral membrane protein	10.1021/jacs.3c03454
**Arf3+GPD complex**	Mouse	P61205	Soluble protein-ligand complex	10.1021/jacs.1c10032
**Mt-VDAC1**	Mouse	Q60932-2	Beta barrel integral membrane protein	Figure S2
**Arf1+GDP**	Mouse	P84078	Soluble protein-ligand complex	10.1021/jacs.1c10032
**CAH2+Zn^{2+}**	Mouse	P00920	Soluble protein-metal complex	10.1021/jacs.1c10032
**SIRT2.2+Zn^{2+}**	Mouse	Q8VDQ8-2	Myelin associated, soluble	Figure S1
**MAL**	Mouse	O09198	Multipass membrane protein	Figure S3
**BASP1**	Mouse	Q91XV3	Membrane-associated via lipid anchor	Figure S4
**CNP1**	Mouse	P16330	Membrane-associated via lipid anchor	Figure S5
**Rab3a+GDP complex**	Mouse	P63011	Membrane-associated via lipid anchor	Figure S6
**Aquaporin-0 tetramer**	Sheep	Q6J8I9	Multipass membrane protein assembly	10.1002/anie.202201458
**γ-crystallin**	Sheep	W5QH67	Soluble, forms oligomers	10.1021/jasms.4c00377
**β-B2-crystallin**	Sheep	W5QCG5	Soluble, forms oligomers	10.1002/anie.202201458
**β-A4-crystallin**	Sheep	W5NUB1	Soluble, forms oligomers	10.1002/anie.202201458

[a]Figure number for MS/MS data or DOI for publication where identified previously.

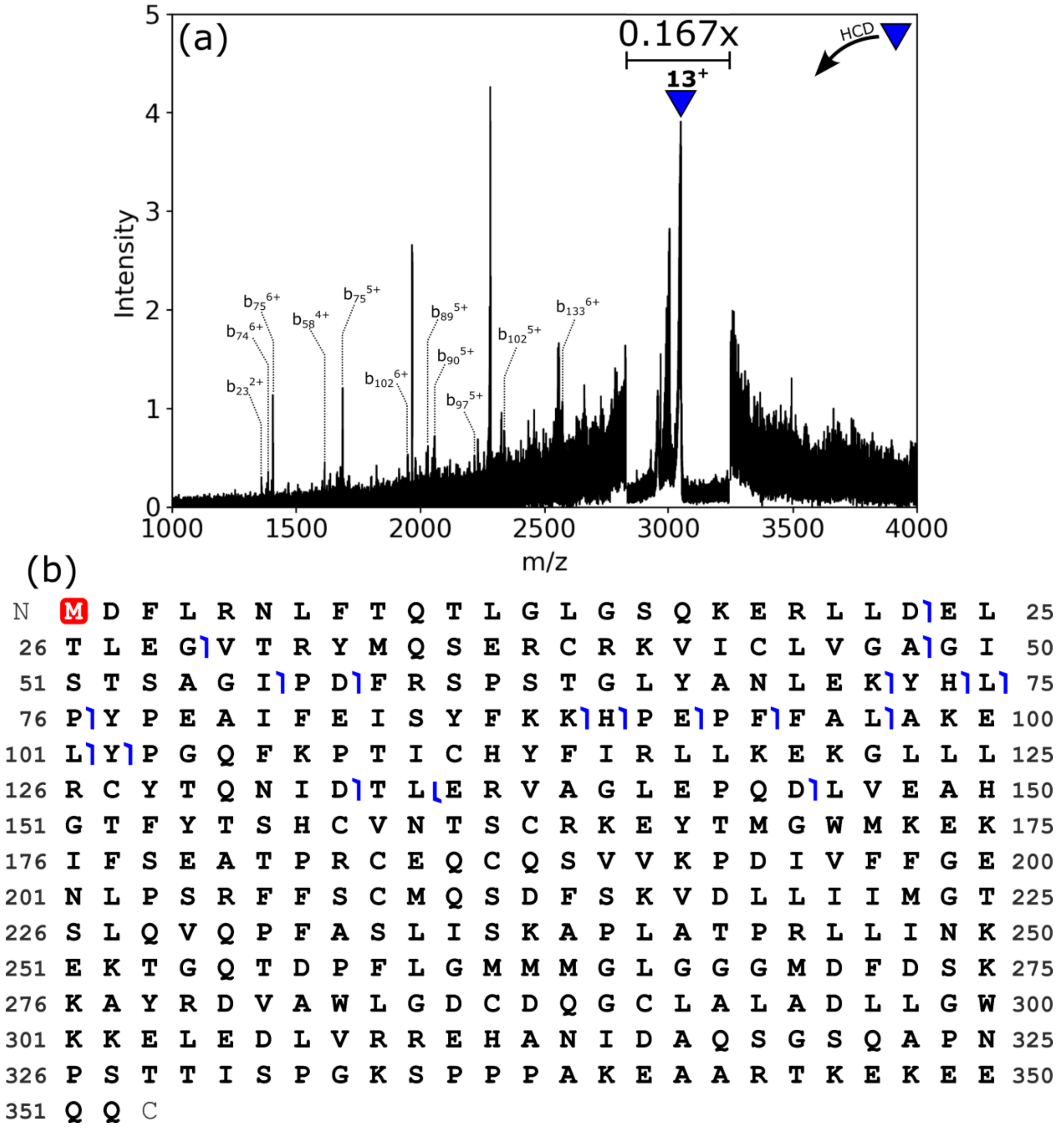

Figure S1: (a) nano-DESI-HCD MS^2 mass spectrum for m/z 3051^{13+}±3.5, NCE = 50% (b) Sequence ion map for SIRT2.2. The N-terminus is acetylated.

Table S2: SIRT2.2 sequence ions.

Ion	Monoisotopic Mass (Da)	Calculated Mass (Da)	Error (ppm)
**b23**	2718.3914	2718.4163	-9.2
**b29**	3362.6817	3362.7387	-17.0
**b48**	5555.7824	5555.8720	-16.1
**b56**	6241.3098	6241.2319	12.5
**b58**	6452.2398	6452.3116	-11.1
**b72**	8016.0098	8016.1161	-13.3
**b74**	8316.1158	8316.2384	-14.7
**b75**	8429.2265	8429.3224	-11.4
**b76**	8524.2959	8524.3752	-9.3
**b89**	10142.1142	10142.2038	-8.8
**b90**	10280.1140	10280.2627	-14.5
**b92**	10505.2518	10505.3580	-10.1
**b94**	10747.4185	10747.4792	-5.6
**b97**	11079.6447	11079.6688	-2.2
**b101**	11523.8112	11523.9275	-10.1
**b102**	11684.8763	11684.9908	-9.8
**b133**	15374.7254	15374.9646	-15.6
**b145**	16684.4780	16684.6320	-9.2

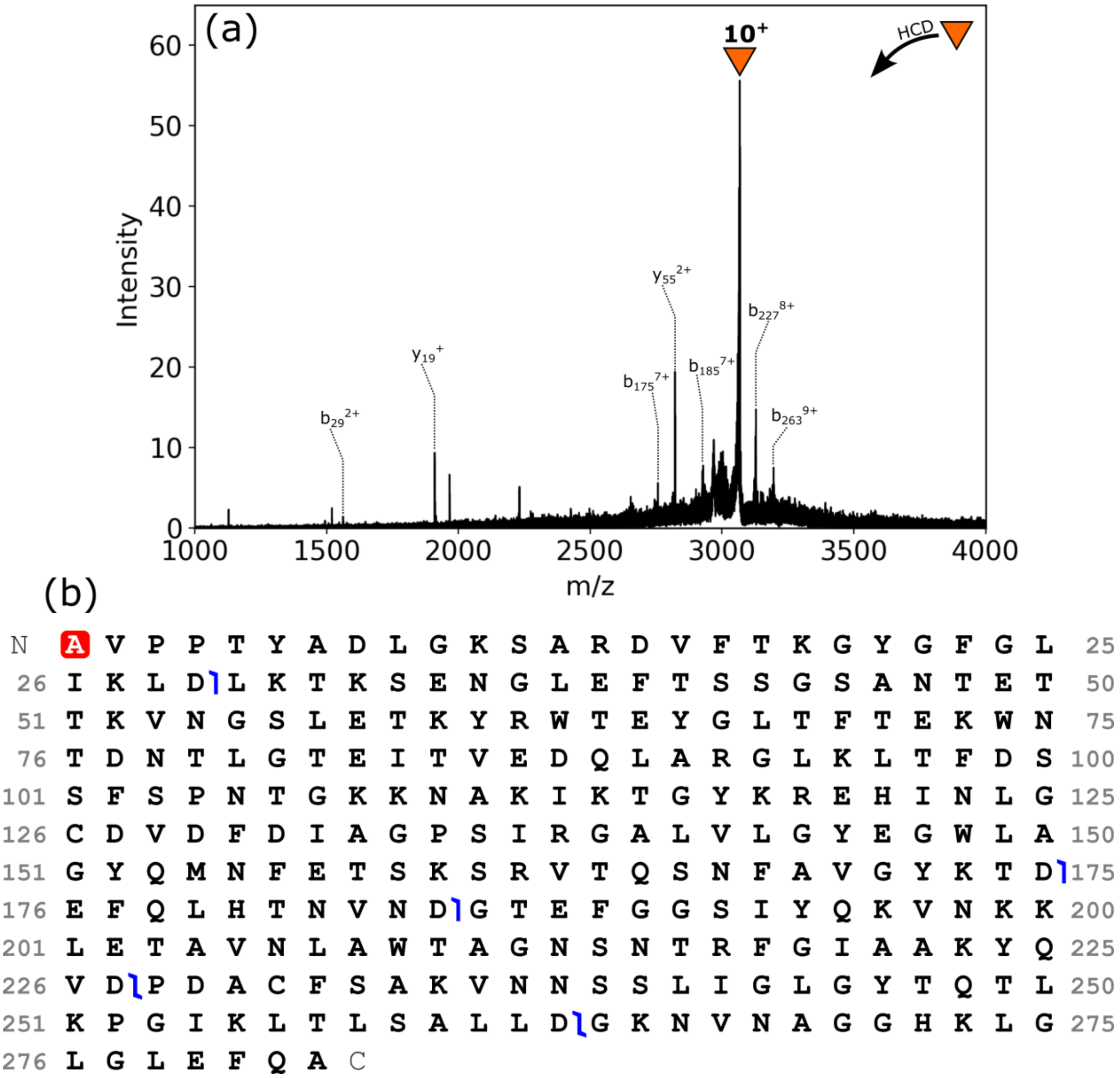

Figure S2: (a) nano-DESI-HCD MS^2 spectrum for mouse Mt-VDAC1, m/z 3067^{10+}±6, NCE = 52% (b) sequence ion map for Mt-VDAC1. The N-terminus is acetylated.

Table S3: Mt-VDAC1 sequence ions.

Ion	Monoisotopic Mass (Da)	Calculated Mass (Da)	Error (ppm)
**y19**	1908.9934	1909.0169	-12.3
**b29**	3122.6065	3122.6634	-18.2
**y55**	5638.9474	5639.0184	-12.6
**y55**	5638.9474	5639.0184	-12.6
**b175**	19282.4880	19282.6879	-10.4
**b185**	20479.9968	20480.2293	-11.4
**b227**	25007.2719	25007.5432	-10.8
**b263**	28738.1937	28738.5446	-12.2

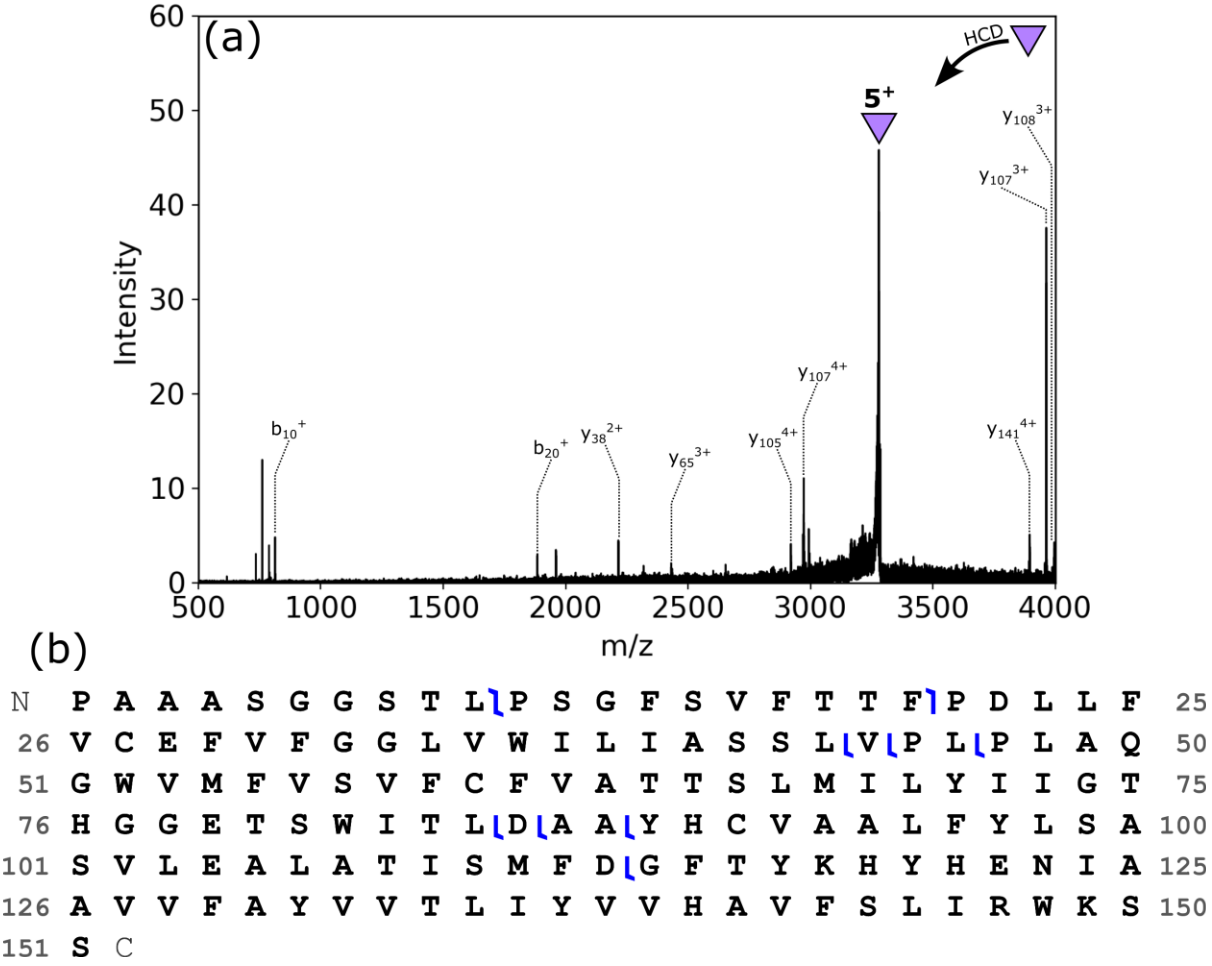

Figure S3: (a) nano-DESI-HCD MS² spectrum of m/z 3279±5, NCE = 45% (b) sequence ion map for mouse MAL. The N-terminus is truncated by two residues (MA-) from the main canonical sequence.

Table S4: sequence ions for MAL.

Ion	Monoisotopic Mass (Da)	Calculated Mass (Da)	Error (ppm)
**b10**	812.3978	812.4028	-6.2
**b20**	1882.8830	1882.9101	-14.4
**y38**	4427.3146	4427.3622	-10.8
**y63**	7144.6425	7144.6903	-6.7
**y65**	7285.6475	7285.7645	-16.1
**y66**	7401.6902	7401.7914	-13.7
**y105**	11669.7378	11669.9623	-19.2
**y107**	11880.9278	11881.0991	-14.4
**y108**	11980.1035	11980.1675	-5.3
**y141**	15568.7888	15569.0286	-15.4

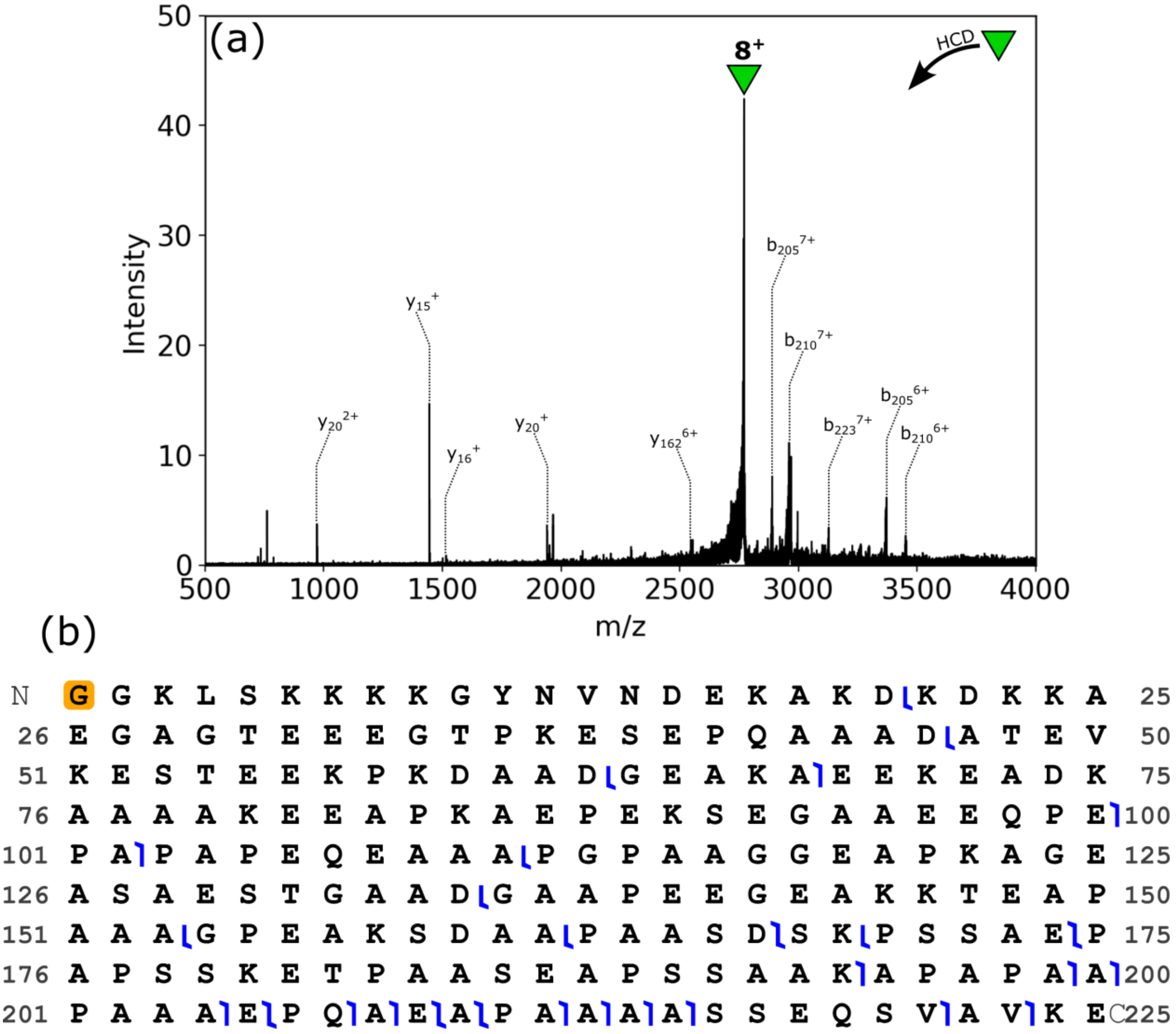

Figure S4: (a) nano-DESI-HCD MS² spectrum of m/z 2771±6, NCE = 40%. (b) sequence ion map for mouse BASP1. The N-terminus is modified with a lipid MW~210 Da for interacting with the cell membrane.

Table S5: sequence ions for BASP1.

Ion	Monoisotopic Mass (Da)	Calculated Mass (Da)	Error (ppm)
**y15**	1443.7058	1443.7205	-10.2
**y16**	1514.7405	1514.7576	-11.3
**y20**	1939.9314	1939.9487	-8.9
**y51**	4721.2671	4721.3259	-12.5
**y56**	5192.4571	5192.5224	-12.6
**y58**	5407.5765	5407.6494	-13.5
**y63**	5849.7814	5849.8354	-9.2
**y72**	6676.1707	6676.2175	-7.0
**b68**	7337.5249	7337.5293	-0.6
**y90**	8354.9543	8355.0336	-9.5
**y114**	10403.8664	10403.9575	-8.8
**b100**	10715.1883	10715.1039	7.9
**b102**	10883.2214	10883.1937	2.5
**y162**	15270.0205	15270.2529	-15.2
**b167**	16742.8911	16742.8995	-0.5
**y179**	17098.9115	17099.1219	-12.3
**b174**	17429.1996	17429.2230	-1.3
**b194**	19294.1594	19294.1397	1.0
**b199**	19701.3221	19701.3565	-1.7
**y205**	19753.3133	19753.3526	-2.0
**b200**	19771.2228	19771.3936	-8.6
**b204**	20081.5873	20081.5577	1.5
**b205**	20211.5291	20211.6003	-3.5
**b207**	20437.6220	20437.7116	-4.4
**b208**	20506.6651	20506.7487	-4.1
**b209**	20636.7654	20636.7913	-1.3
**b210**	20708.7857	20708.8284	-2.1
**b212**	20876.7800	20876.9183	-6.6
**b213**	20945.8272	20945.9554	-6.1
**b214**	21016.9195	21016.9925	-3.5
**b215**	21088.9963	21089.0296	-1.6
**b221**	21706.2885	21706.2953	-0.3
**b223**	21876.3016	21876.4008	-4.5

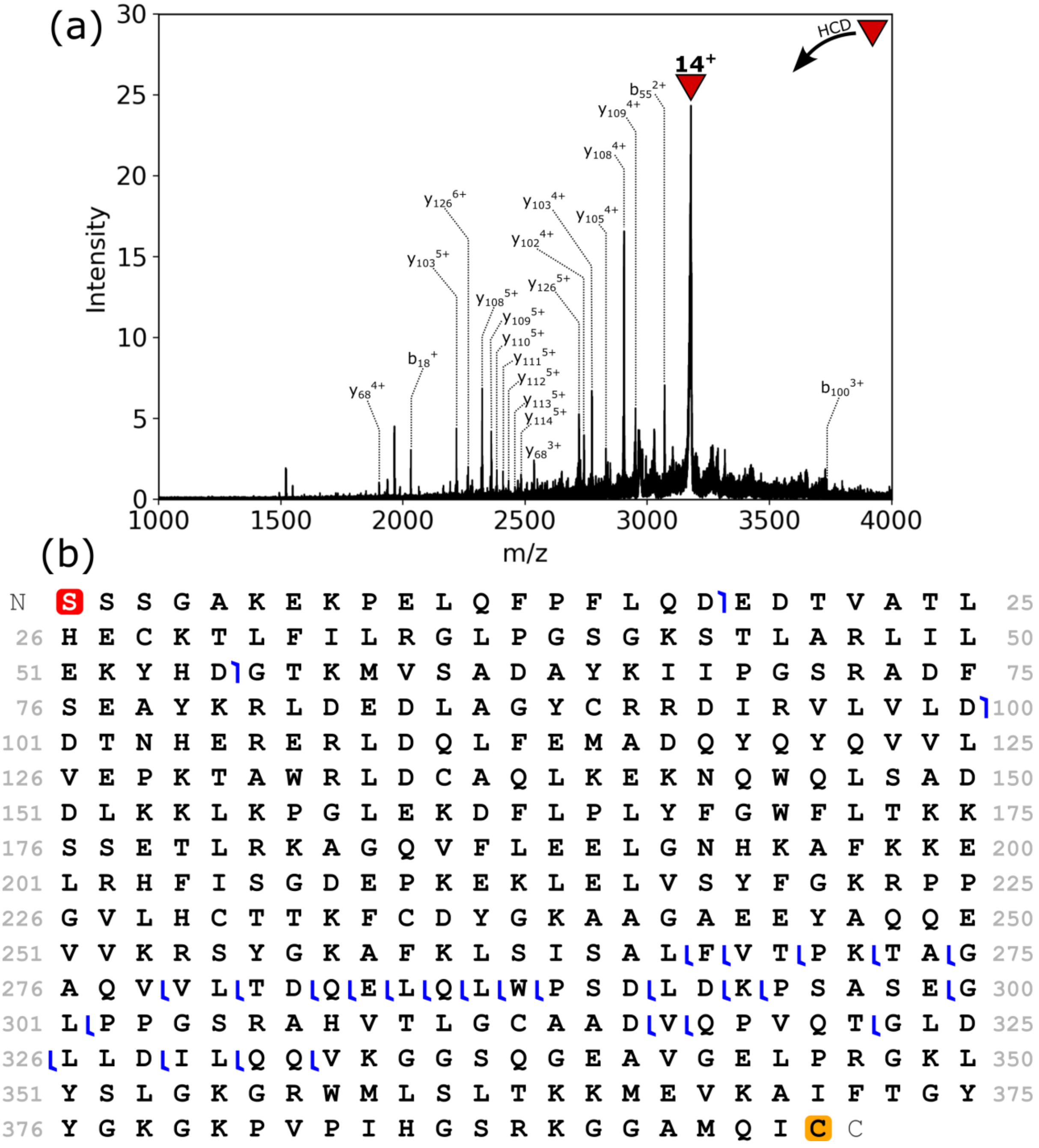

Figure S5: nano-DESI-HCD MS² mass spectrum for m/z 3181.4^{14+}±7.5, NCE = 58%. (b) Sequence ion map for CNP1. The N-terminus is acetylated, the C-terminus is truncated by three residues (-TII) from the canonical sequence, and the C-terminal cysteine residue is modified with an acyl chain with MW ~286 Da.

Table S6: sequence ion table for 2',3'-cyclic-nucleotide 3'-phosphodiesterase (CNP1).

Ion	Monoisotopic Mass (Da)	Calculated Mass (Da)	Error (ppm)
**b18**	2030.9641	2030.9843	-10.0
**b55**	6137.1075	6137.1829	-12.3
**b100**	11194.6268	11194.7684	-12.6
**y64**	7120.8689	7120.7589	15.4
**y66**	7376.9548	7376.8761	10.7
**y68**	7603.1260	7603.0442	10.8
**y68**	7603.1470	7603.0442	13.5
**y71**	7944.3038	7944.2393	8.1
**y74**	8229.4590	8229.3717	10.6
**y79**	8782.7434	8782.6577	9.8
**y80**	8881.7584	8881.7262	3.6
**y95**	10314.4689	10314.4143	5.3
**y95**	10314.5083	10314.4143	9.1
**y97**	10484.5752	10484.5198	5.3
**y97**	10484.5924	10484.5198	6.9
**y102**	10955.8100	10955.7163	8.6
**y103**	11083.8297	11083.8113	1.7
**y103**	11083.8743	11083.8113	5.7
**y105**	11311.9372	11311.9223	1.3
**y105**	11311.9618	11311.9223	3.5
**y105**	11311.9714	11311.9223	4.3
**y108**	11611.0946	11611.0340	5.2
**y108**	11611.1077	11611.0340	6.3
**y109**	11797.0977	11797.1133	-1.3
**y109**	11797.1214	11797.1133	0.7
**y109**	11797.1715	11797.1133	4.9
**y110**	11910.2108	11910.1974	1.1
**y111**	12038.2478	12038.2559	-0.7
**y112**	12151.3383	12151.3400	-0.1
**y113**	12280.4119	12280.3826	2.4
**y114**	12408.4766	12408.4412	2.9
**y116**	12624.5776	12624.5158	4.9
**y118**	12836.6251	12836.6683	-3.4
**y122**	13191.8972	13191.8538	3.3
**y124**	13363.9574	13363.9386	1.4
**y126**	13589.0621	13589.0863	-1.8
**y126**	13589.0886	13589.0863	0.2
**y128**	13789.2383	13789.2024	2.6
**y129**	13936.2881	13936.2708	1.2

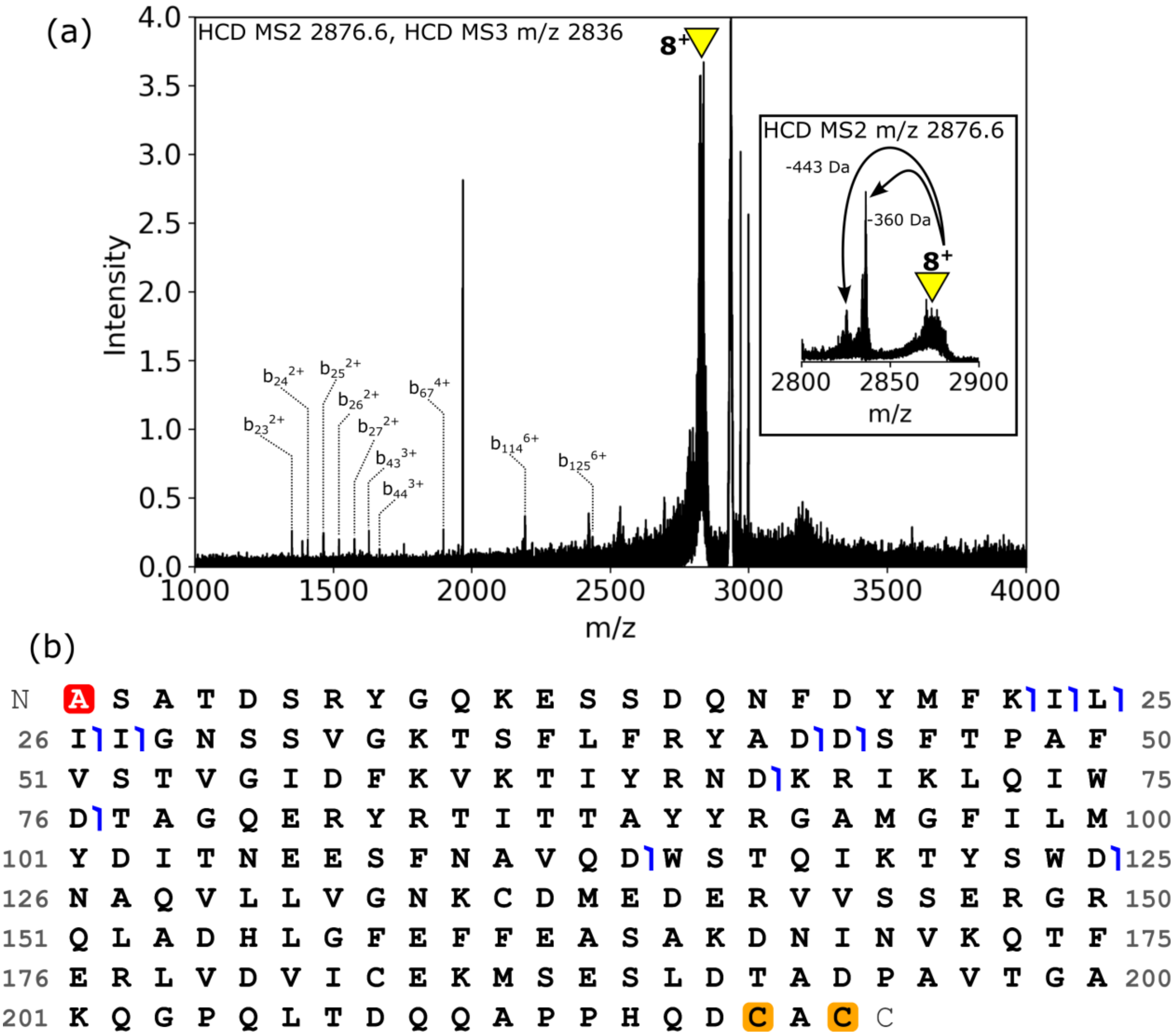

Figure S6: (a) nano-DESI-HCD MS^n of Rab3a. The inset shows HCD MS^2 dissociation of the GDP ligand from Rab3a+GDP complex at m/z 2876.6^{9+}±6, NCE = 40% the dominant dissociation pathway. Product ions including apo-Rab3a (~m/z 2827.5^{9+}) were isolated m/z 2836.5±20 and fragmented by HCD MS^3 with NCE = 48% to generate sequence ions. (b) The sequence of Rab3a with sequence ions labelled. The N-terminus is acetylated (red) and both C-terminal cysteine residues are geranylgeranylated (orange).

Table S7: sequence ions for Rab3a.

Ion	Monoisotopic mass (Da)	Calculated mass (Da)	Error (ppm)
**b23**	2698.1410	2698.1605	-7.3
**b24**	2812.2284	2812.2519	-8.4
**b25**	2925.3128	2925.3360	-7.9
**b26**	3037.3907	3037.4127	-7.2
**b27**	3150.4792	3150.4968	-5.6
**b43**	4879.3049	4879.3391	-7.0
**b44**	4995.3097	4995.3661	-11.3
**b67**	7581.6256	7581.7143	-11.7
**b76**	8763.3558	8763.4223	-7.6
**b114**	13139.3273	13139.4693	-10.8
**b125**	14533.9217	14534.1152	-13.3

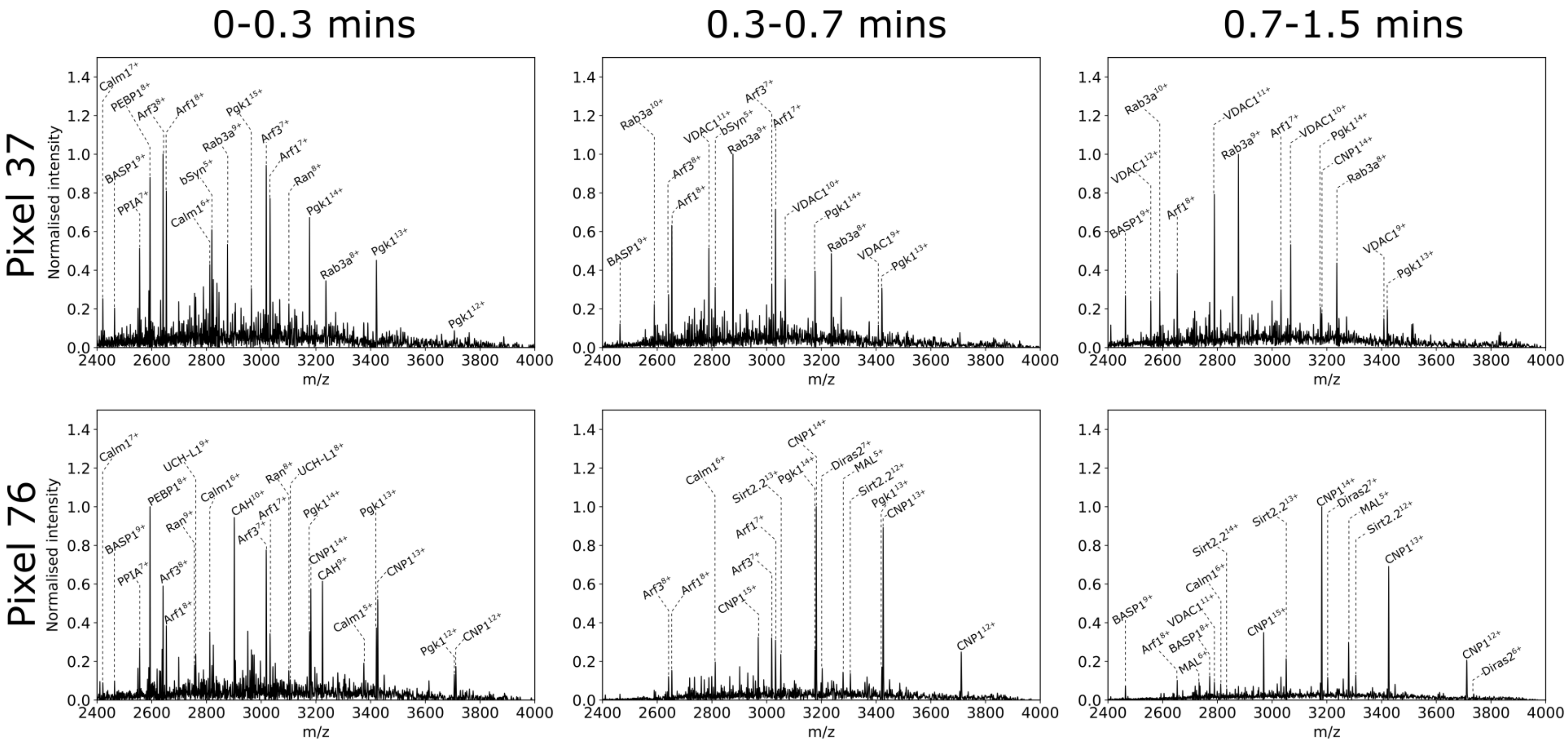

Figure S7: representative mass spectra for three elution time ranges from two different pixels representing grey (pixel 37) and white (pixel 76) brain matter.

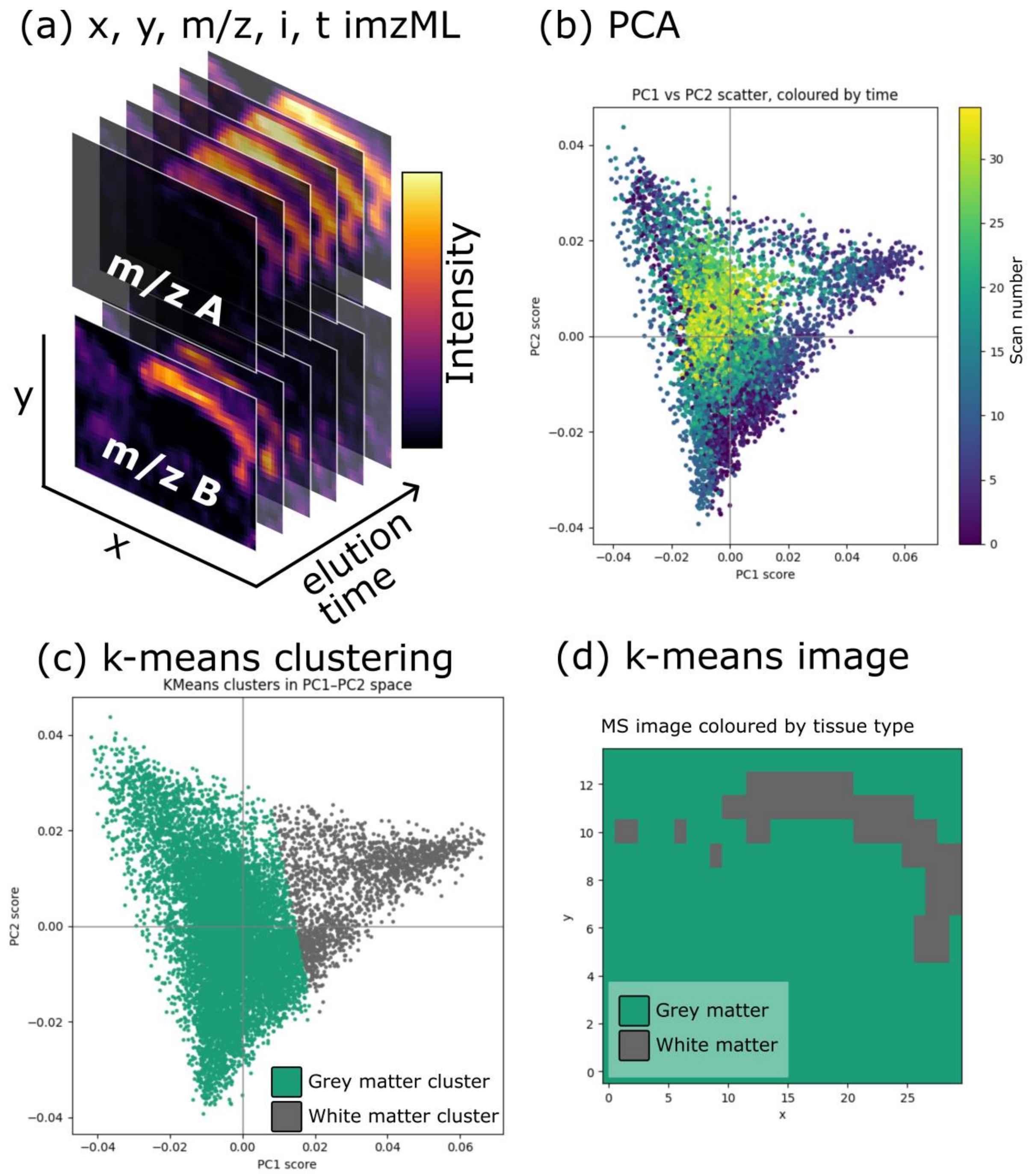

Figure S8: (a) representation of the imzML file containing spatial (x, y), m/z, intensity (i) and elution time (t) dimensions. (b) Principal component analysis score plot for PCs 1 (explained variance ~ 13%) and 2 (explained variance ~9%) from the imzML file. Additional PCs did not provide variance explanation > 5%. Each data point represents a mass spectrum at a single time point (50% of the total data points were plotted). (c) Data points for grey and white matter were separated by k-means clustering (input PCs = 1, 2. Clusters = 2). (d) Map of the imzML pixels according to their k-means cluster. The corpus callosum (white matter) is distinct among the bulk grey matter.

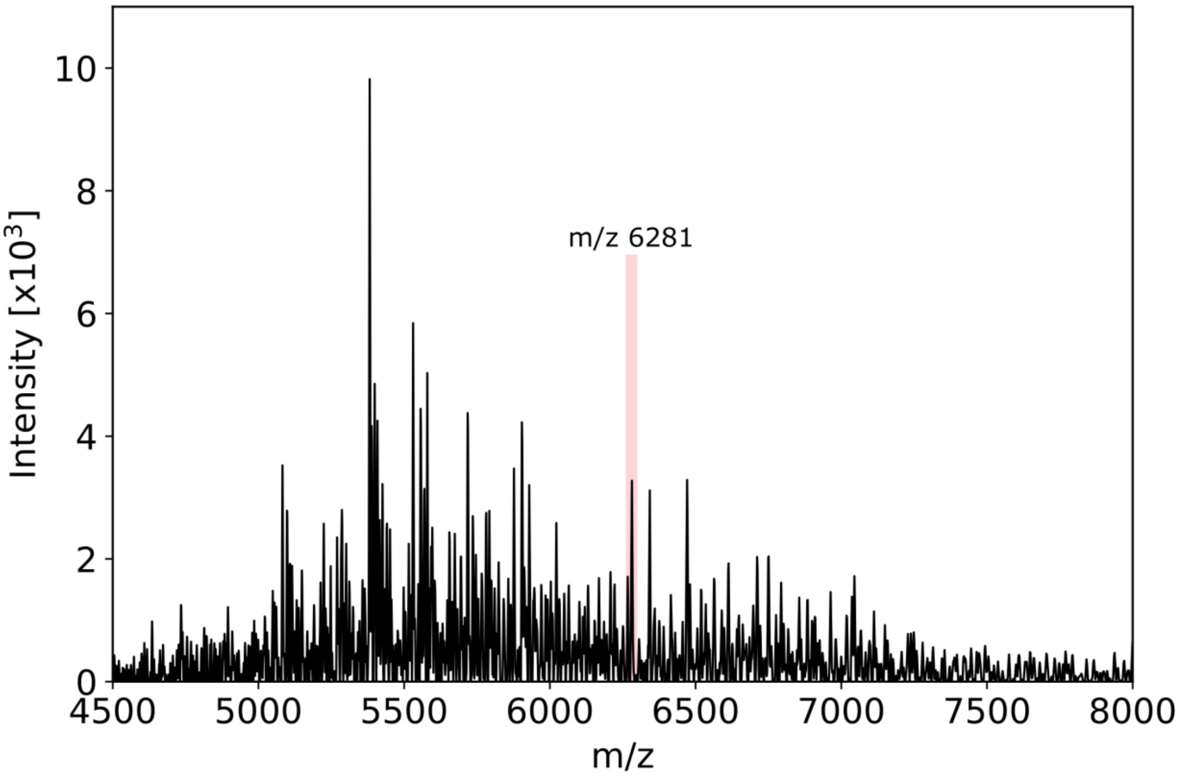

Figure S9: nano-DESI full scan mass spectrum of protein complexes in the eye lens in the range m/z 4500 – 8000. The signal at m/z 6281 that was subsequently targeted for MS/MS is labelled.

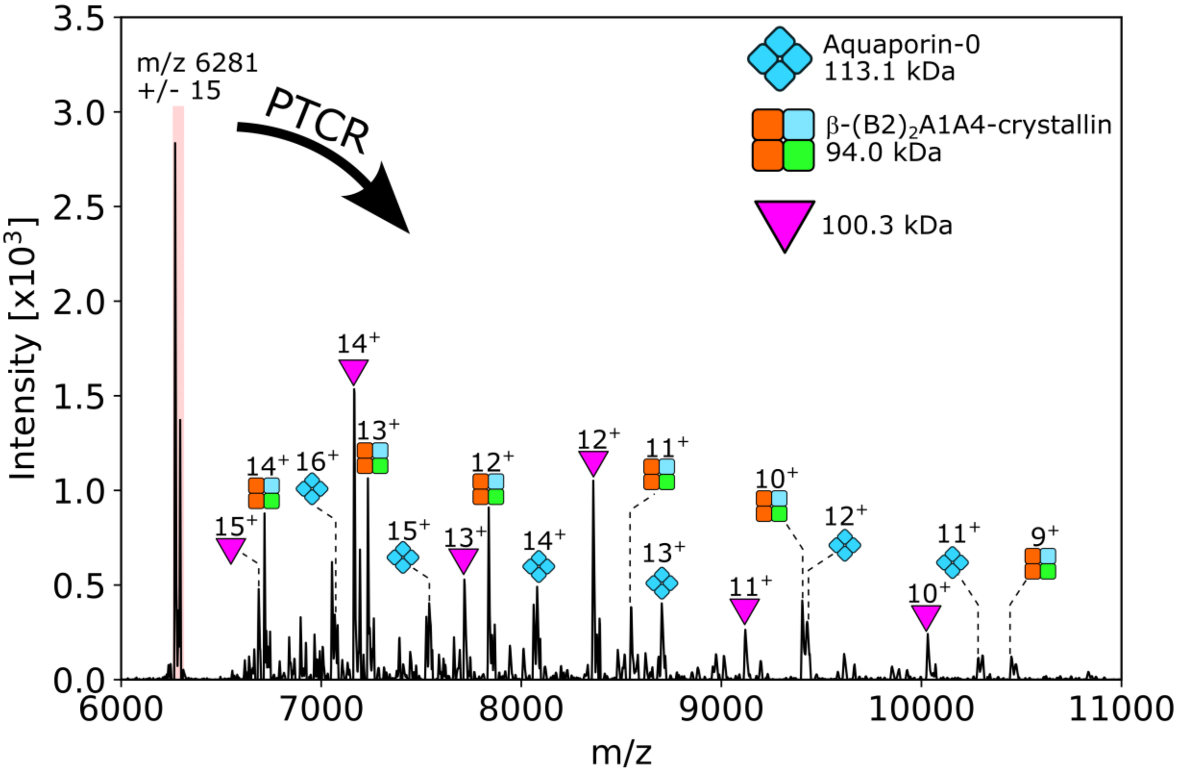

Figure S10: nano-DESI-PTCR MS² of m/z 6281±15, reaction time = 5 ms. Product ions indicated at least three proteins and/or complexes between 90 – 115 kDa in the isolation window. Two of these protein complexes were previously identified.[1]

(1) Hale, O. J.; Cooper, H. J. Native Ambient Mass Spectrometry of an Intact Membrane Protein Assembly and Soluble Protein Assemblies Directly from Lens Tissue *Angewandte Chemie-International Edition* **2022**, *61*, e202201458, ARTN e202201458

10.1002/anie.202201458
